# Experimental realization of an open cavity

**DOI:** 10.1038/srep05965

**Published:** 2014-08-06

**Authors:** Xiaochen Ge, Sailing He

**Affiliations:** 1 State Key Laboratory of Modern Optical Instrumentations, Centre for Optical and Electromagnetic Research, JORCEP, Zhejiang University, Hangzhou 310058, China; 2 Department of Electromagnetic Engineering, School of Electrical Engineering, Royal Institute of Technology, S-100 44 Stockholm, Sweden

## Abstract

The design and experimental demonstration of an open cavity in the microwave region is presented. The resonance condition is achieved through the cancellation of lightpaths in positive and negative refractive index materials. The positive index material is a structured aluminium surface supporting a spoof surface plasmon mode, and the negative index material is a photonic crystal made of alumina. A resonance peak is observed in the measured spectrum at which the electric field distribution agrees with numerical simulation.

Driven by potential applications in superlenses and other novel phenomena^1^,^2^,^3^, negative refraction photonic crystals have been extensively studied^3^,^4^,^5^. Generally there are two principles of operation for achieving negative refraction photonic crystals. One exploits the negative group velocity near the centre of the Brillouin zone (Γ) where the phase and group velocities have opposite signs. Since the equal-frequency contour (EFC) is near-circular, an effective index *n*_eff_ can be defined, so that the photonic crystal behaves like an isotropic, uniform, left-handed material in a specific frequency range^4^. The other method uses the band structure at the edge of the Brillouin zone where the EFC is convex^6^. There is no negative effective index to be defined in this case. A similar behaviour can also be found at the so-called “spoof plasmon” surface waves on structured metal surfaces^7^.

One particular application for photonic crystals with a negative effective index is an open cavity, a cavity without reflecting walls. In a ray-tracing view, the resonant condition is achieved by arranging alternating positive-index and negative-index material wedges with minimal interface-reflection so that the positive and negative lightpaths cancel each other out and form an open cavity with high quality factor^8^,^9^,^10^,^11^. Open cavities are promising in applications such as sensing or lasing where strong light-matter interaction can be achieved by the large overlap of the resonant mode and the material of interest (measurands, gain materials, etc.). However, to the best of our knowledge, there has still been no experimental realization of open cavities in either the microwave or optical region. In this work, we will experimentally demonstrate a microwave open cavity with a hybrid of photonic crystal and spoof plasmon structure.

## Results

**Figure 1:**
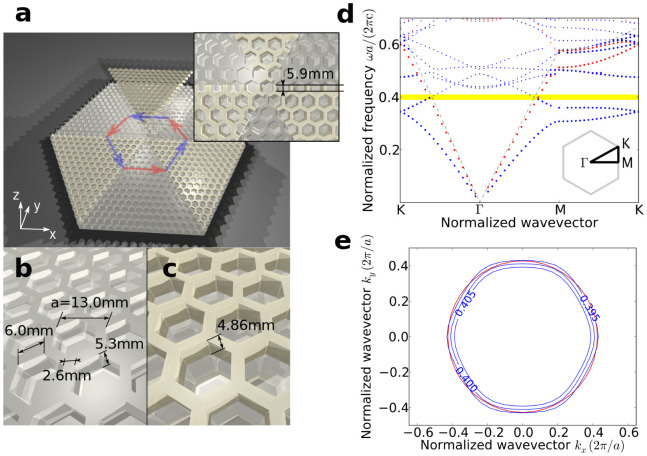
Modeling and band structures of the designed open cavity. (a) Perspective view of the designed open cavity. One piece of the dielectric slabs is raised a bit to show the underneath metal structure. The inset shows the centre of the cavity and the interface between the positive and negative index materials. Red and blue arrows are the lightpaths in positive and negative index materials, giving a ray-tracing view. (b) Schematic diagram for the positive index structure. (c) Schematic diagram for the negative index structure. (d) Band diagram for positive index (red) and negative index (blue) unit cells. The gray dashed curve is the position of the light cone for air. The yellow area is where the two bands overlap. The small dots are the modes in the light cone. Γ, M, K are high symmetry points in the irreducible Brillouin zone of the hexagonal lattice, as shown in the inset. (e) EFCs of the positive index (red) and negative index (blue) bands in the overlapping area. The normalized frequency for the positive index band curve is 0*.*400 c/*a*, and the normalized frequencies for the negative index band are indicated in the figure.

The designed open cavity is shown in Figure 1(a). A triangular lattice of hexagonal holes drilled on a perfect metal surface is used as the positive index material. The dimensions of the structure are shown in Figure 1(b). In addition to the structure shown in Figure 1(a), a metal cover is suspended over the open cavity. Its purpose will be explained later. For the convenience of fabrication and measurement, the lattice constant is chosen to be *a* = 13.0 mm. From the calculated TM (electric field polarized perpendicularly to the metal surface) band diagram in Figure 1(d) it can be seen that a spoof plasmon surface mode with an effective index slightly above 1 is supported. The negative index material, as shown in Figure 1(c), has the same structure and dimensions except that a dielectric (alumina, *ε* = 9*.*0) photonic crystal layer with hexagonal holes is placed on top of the metal surface. The band diagram of the negative index material is plotted in Figure 1(d) as blue dots. It can be seen that the second band is pulled down by the presence of the dielectric structure and overlaps with the first band of the positive index material at the normalized frequency around 0*.*4 c*/a* (c is the speed of light in vacuum.). The EFCs of the bands in the region of overlapping frequencies (shown in Figure 1(e)) is near isotropic, indicating that an effective index can be defined in that frequency window. Also the group velocity (the gradient of the band diagram) is in the opposite direction of the wave vector, indicating that the defined effective index is negative.

**Figure 2:**
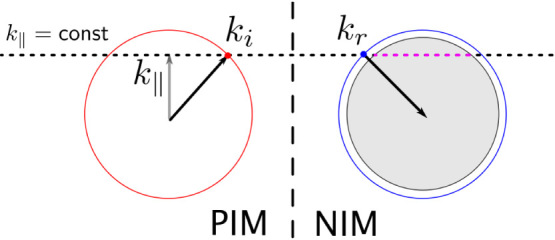
Schematic diagram for the refraction process at the interface. The red circle is the EFC of an isotropic positive index material, and the blue curve is the EFC of an isotropic negative index material. The vertical dashed line is an indication for the interface of the two materials. The horizontal dotted line is the locus of the tip of the wavevectors with a constant parallel component. The grey solid disk is the cross section of the air cone at the given frequency. The purple dashed line is the modes in the air cone that may be excited during the refraction process.

Although both structures support surface modes with matching effective indices with opposite signs, the coupling between these modes and the existence of open cavity resonance are not guaranteed. The symmetries of the modes must be matched to achieve a high transmission^9^. Also, the continuous modes in the air light cone have to be taken into consideration. Figure 2 shows the negative refraction process at the interface of the positive index material (PIM) and the negative index material (NIM). When refraction occurs, the parallel component of the wavevector is conserved. Besides the negative index mode on the EFC (blue dot), the leaky modes in the air light cone (purple dashed line) also satisfy this condition. These modes have *ω > k* and will propagate to the free space. Since the positive index mode exhibits a behaviour largely resembling that of a plane wave, these leaky modes may also be excited alongside the negative refraction mode provided that the mode matching and symmetry requirements are met. Thus the loss at the refraction interface is large. After applying a metal cover above the structure, the space between the grooved metal surface and the bottom surface of the metal cover can be considered to be a plate waveguide. Thus in the region where *ω > k*, only the discrete waveguide modes rather than the continuous free space modes can exist, as indicated by the small dots in Figure 1(d). By adjusting the parameters the band structure can be tuned so that in the frequencies of interest the positive effective index modes can only couple to the negative effective index modes, as indicated in the yellow overlapping area in Figure 1(d). The position of the termination of the lattice at the interface of the two materials also influences the transmission^10^,^12^. Thus the cells at the edge of the dielectric wedges are cut at a specific position, as illustrated in the inset of Figure 1(a). The gap height *h* between the metal surface and the metal cover is fixed to be 0*.*51 *a = * 6.63 mm.

**Figure 3:**
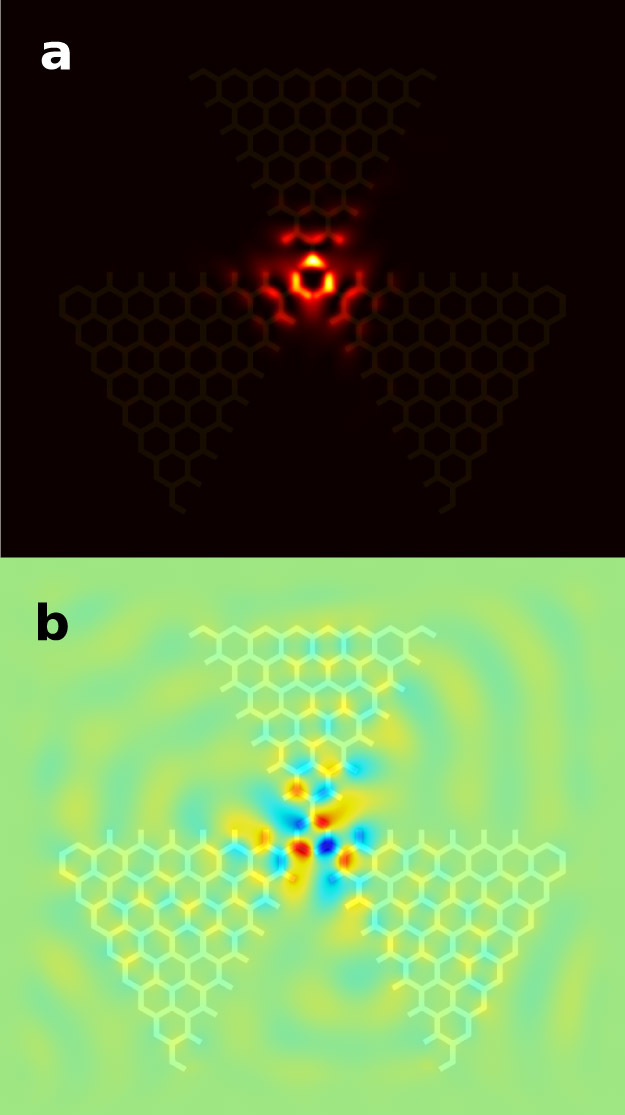
Simulated field distributions of the resonant mode at the top surface of the dielectric slabs, overlaid with the dielectric structure. (a) Electric field intensity |*E*|^2^. (b) A time snapshot of electric field *E_z_*.

The simulated field intensity is shown in Figure 3(a). The frequency and quality factor of the mode are extracted^13^ to be *f* = 0*.*4030 c*/a*, which corresponds to 9*.*294 GHz, and *Q* = 814. The resonant frequency is not at the exact value where the absolute values of the positive and negative effective index are equal to each other. This may be caused by the tuning of the photonic crystal lattice termination at the interface, which slightly modifies the light path and influences the phase. The number of periods of the photonic crystal has a minor influence on the quality factor of the mode, which is understandable since the field is largely confined to around the cavity centre.

**Figure 4:**
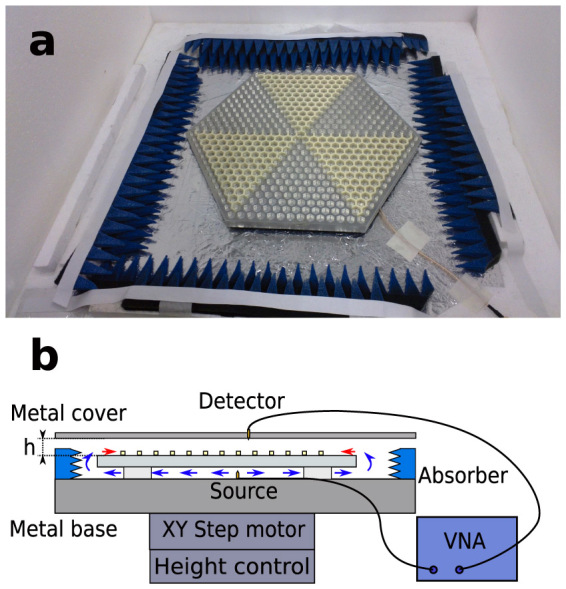
Fabricated sample and experimental setup. (a) A photograph of the fabricated sample with the surrounding absorber and the cable connecting the source probe (below the sample). The detector probe and the metal cover are not shown in the picture. (b) Side view of the experimental setup. The blue and red arrows indicate the waveguide mode and the surface mode excited at the edge of the aluminium plate.

**Figure 5:**
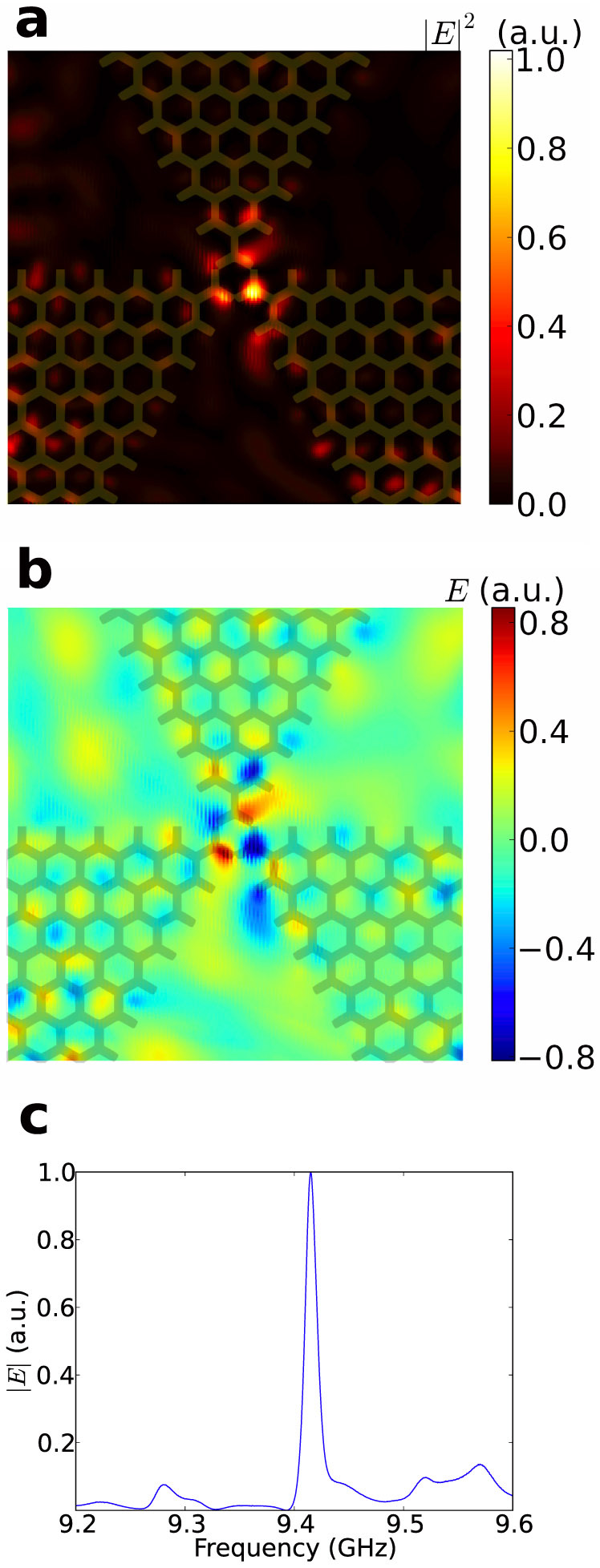
Measured resonance. (a) Electric field intensity at the resonance frequency, overlaid with the dielectric structure. The position of the detector is on the bottom surface of the metal cover. (b) Electric field at a specific phase, overlaid with the dielectric structure. (c) Measured spectrum near the centre of the cavity.

Figure 4 shows the fabricated sample and the experimental setup. Details for the experiment can be found in the “Methods” section. Since the measured parameter *S*_21_ is proportional to the electric field, the measured electric fields are plotted as *S*_21_ with an arbitrary unit. The measured intensity of the electric field is shown in Figure 5(a) and the measured electric field at a specific phase is shown in Figure 5(b) (the calculated field in Figure 3(b) is at a time snapshot chosen to match Figure 5(b)). It can be seen that the electric field concentrates around the center, showing a pattern similar to the calculated results in Figure 3, indicating the existence of a resonance mode. The spectrum of the field at a position near the cavity centre is measured and plotted in Figure 5(c), where a resonance peak is clearly seen. The frequency of the peak is 9*.*415 GHz and the full-width half maximum (FWHM) of the peak is 0*.*013 GHz. Hence, the quality factor of the mode is calculated to be *f/*Δ*f* = 724. The differences of the calculated and measured frequencies and quality factors could be caused by error in assembling the sample, error in the parallelism between the sample and the metal cover, and the difference between realistic and theoretical material permittivities. Also the measured intensity has an asymmetrical pattern compared to that of the calculated result, which could be caused by the errors mentioned above breaking the symmetry of the structure.

## Discussion

**Figure 6:**
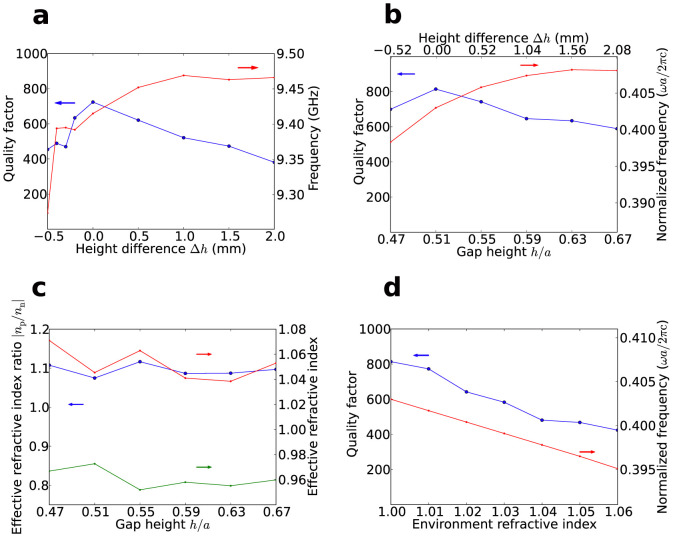
Plots of resonance modes and effective indices with respect to varying gap height and the sensing capability of the open cavity. (a) Measured mode frequencies (red) and quality factors (blue). (b) Simulated mode frequencies (red) and quality factors (blue). (c) Calculated positive effective index (*n*_p_, red), negative effective index (|*n*_n_|, green) and their ratio (|*n*_p_*/n*_n_|, blue) at the respective resonant frequencies. (d) Simulated resonant frequencies (red) and quality factors (blue) with varying environment refractive index.

To verify that the resonance is indeed caused by the cancellation of the light paths in the positive and negative index materials, we change the effective indices of the positive and negative index materials by tuning the gap height *h*. A series of measurements with varying values of *h* were performed. The measured mode frequencies and quality factors are plotted in Figure 6(a). Since the precise value of *h* is not obtainable in our setup, the x-axis of the plot is the difference of the gap height Δ*h* with respect to the optimal height that gives a mode with the largest quality factor (the measured results shown above are for this case). The figure shows that the resonant frequency increases with increasing values of *h*, and the quality factor drops gradually while *h* deviates from the optimal value. A similar trend can be seen in the simulated result in Figure 6(b). The increasing resonant frequency with increasing *h* can be understood by considering the sample together with the top cover as an MIM (metal-insulator-metal) plasmonic waveguide, and the mode as the fundamental TM_0_ mode that has no cut-off thickness^14^,^15^. Increasing the gap height causes the coupling between the two metal/dielectric interfaces to weaken, and the TM_0_ mode would approach the surface mode of the structure without the presence of the metal cover, which has a higher frequency. From Figure 6(c) it can be seen that the effective index ratio of the positive and negative index materials at the resonant frequencies stays nearly constant for varying gap heights. Consider the condition for resonance *n*_p_*L*_p_ + *n*_n_*L*_n_ = 0 where *n*_p_, and *n*_n_ are the effective indices and *L*_p_, *L*_n_ are the lengths of physical paths for positive and negative index materials, indicated in Figure 1(a) as red and blue arrows. Since the planar structure is fixed, the ratio *L*_p_*/L*_n_ is thus fixed. Therefore resonance can only occur at the frequency at which the ratio of the indices satisfies the above condition. The quality factor of the open cavity mode depends on the reflectance at the positive and negative index material interface. Therefore, only the resonant frequency at which the reflection is low could have a high quality factor, and the quality factor degrades for other resonant frequencies. The refractive index sensing capability of the designed device is also explored. Consider a case when the open cavity is immersed in the measurand, and let *h* be fixed at the optimal value. The simulated resonance frequencies and quality factors with a varying environment refractive index are shown in Figure 6(d). Given the period *a* = 13.0 mm, the sensitivity is calculated to be 2*.*998 GHz*/*RIU at the frequency 9*.*294 GHz.

In conclusion, we designed an open cavity with alternating positive and negative index materials for spoof plasmon surface waves in the microwave region. The experimental results agree well with simulations. Note that the open cavity experiment we have demonstrated here is to show the “openness” of the cavity on the horizontal plane. The condition for open cavity resonance and the sensing capability of the device have also been discussed. The open cavity has a good sensitivity due to the large overlap of the resonance mode and the measurand. The designed open cavity works in the microwave region. However, the working frequency can be scaled to terahertz or optical regions, where potential sensing applications are expected. Due to the need of suppressing the leaky wave, in the terahertz region the approach of employing a metal cover in this work may still be applicable; while in the optical region, metallic structures will have some difficulty due to the increasing material loss. Instead, thick dielectric structures may be fabricated by lithography and deep etching techniques to create a 2D-equivalent optical device. Exploring the negative refraction in 3D photonic crystals^16^ may be another viable approach with the development of the fabrication techniques^17^.

## Methods

### Simulation

The band diagram, EFCs and resonance modes are calculated by the 3D finite-difference time domain method. A freely available FDTD package^18^ is used for the simulations performed in the present work.

### Sample fabrication

The material of the metal part of the sample is aluminium and that of the dielectric part is 99.6% pure alumina. Those parts were machined separately on CNC (computer numerical control) mills and assembled afterwards.

### Measurement

As shown in Figure 4, foam blocks (*ε* ≈ 1) are put under the sample to suspend it. The source probe, which acts as a point source, is fixed at the back side of the sample. The precise position of the source does not affect the measurement. A detector probe penetrates through a hole in the metal cover. The source probe excites the electromagnetic wave in the plate waveguide formed by the back side of the aluminium plate and the top surface of the metal base. The waveguide mode propagates to the edges of the aluminium plate and excites the spoof plasmon mode there, as indicated by the arrows in Figure 4(b). This setup avoids the interference from the point source since the signal from the source does not propagate to the detector directly. The metal cover is in a fixed position and the sample is mounted at a motor-controlled stage. The distance between the sample and the metal cover can be controlled by raising or lowering the stage. The source and detector probes are connected to the two ports of a vector network analyzer. The stage step motor is programmed to scan over the sample area, and the magnitude and phase of the forward transmission (*S*_21_) between the two ports over a frequency range at each position are measured.

## Author Contributions

S.H. initiated the idea and supervised the finding of the present work. X.G. did the calculation and experiment. X.G. and S.H. wrote the paper.
